# Lung organoids as a human system for *Mycobacteria* infection modeling and drug testing

**DOI:** 10.1111/febs.70265

**Published:** 2025-09-21

**Authors:** Stephen Adonai Leon‐Icaza, Romain Vergé, Raoul Mazars, Laurence Berry, Céline Cougoule

**Affiliations:** ^1^ Institut de Pharmacologie et Biologie Structurale (IPBS) Université de Toulouse, CNRS, UPS France; ^2^ Laboratory of Pathogen Host Interactions (LPHI) Université Montpellier, CNRS France

**Keywords:** airway defense mechanisms, host‐directed therapies, infection modeling, lung organoids, *Mycobacteria*

## Abstract

Mycobacterial infections remain a global public health challenge. Each year, high rates of morbidity and mortality worldwide are a consequence of chronic respiratory infections due to *Mycobacteria*. According to the World Health Organization (WHO), in 2023, 10.8 million individuals fell ill with *Mycobacterium tuberculosis* (Mtb), resulting in an estimated 1.25 million deaths. This positions tuberculosis (TB) as the leading cause of death from a single pathogen worldwide after the coronavirus disease (COVID‐19) pandemic. On the other hand, the cases of people affected by nontuberculous mycobacteria (NTM) have risen globally, but the precise incidence and prevalence of both pulmonary and extrapulmonary disease remain unknown. In Europe, nontuberculous mycobacterial pulmonary diseases affect between 0.2 and 2.9 per 100 000 individuals, mainly patients with cystic fibrosis (CF) and non‐CF bronchiectasis. The diagnosis and treatment of mycobacterial infections are challenging and complex, frequently requiring long‐duration treatments with several antibiotics, which in most cases leads to poor patient outcomes. As the role of immune cells has been extensively assessed, in this Review, we summarize the current knowledge about the contribution of epithelial cells in the early steps of *Mycobacteria* infections. Additionally, we describe how human lung organoid technology provides new tools to better understand host–*Mycobacteria* interactions in the airways and test new therapeutic targets.

## Graphical abstract

Mycobacterial infections, including tuberculosis (TB) and infections by nontuberculous *mycobacteria* (NTM), are still public health issues. In 2023, TB caused 1.25 million deaths, while NTM remain a clinical challenge for patients with cystic fibrosis (CF). This Review explores the role of lung epithelial cells in the early stages of *Mycobacteria* infections and describes how lung organoids constitute relevant tools to understand host–pathogen interactions and test therapeutic compounds. Created in https://BioRender.com.

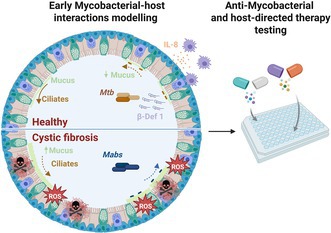

**Abbreviations**
3D: three‐dimensional
AEC1: alveolar type 1 epithelial cells
AEC2: alveolar type 2 epithelial cells
ALI: air–liquid interface
AM: alveolar macrophages
AMPs: antimicrobial peptides
AMR: antimicrobial resistance
ASCs: adult stem cells
BCG: Bacillus Calmette–Guérin
CF: cystic fibrosis
CFTR: cystic fibrosis transmembrane conductance regulator
COPD: chronic obstructive pulmonary disease
COVID‐19: Coronavirus disease
DC: dendritic cells
EC: airway epithelial cells
ESC: embryonic stem cells
GPLs: glycopeptidolipids
HNEO: human nasal epithelial organoids
iPSCs: induced pluripotent stem cells
Mabs: *Mycobacterium abscessus*
MHC‐II: antigen presentation molecules of class II
Mtb: *Mycobacterium tuberculosis*
NHBE: normal human bronchial epithelial cells
NTM: nontuberculous mycobacteria
PCL: periciliary liquid layer
PSCs: pluripotent stem cells
R: rough
ROS: reactive oxygen species
S: smooth
TB: tuberculosis
TLRs: Toll‐like receptors
WHO: World Health Organization

## Introduction

Tuberculosis (TB) remains the leading cause of death due to a single pathogen, *Mycobacterium tuberculosis* (Mtb). Moreover, there has been a remarkable rise in infections with closely related nontuberculous mycobacteria (NTM), including *Mycobacterium abscessus* (Mabs), which pose a particular threat to patients with chronic pulmonary disease such as cystic fibrosis (CF) [1]. Despite the Bacillus Calmette–Guérin (BCG) vaccine, unfortunately, it has been ineffective at reducing the global TB burden [2, 3]. The standard of care for drug‐sensitive TB is a 6‐month regimen with four antibiotics, with a situation equally challenging for patients with NTM infections. Indeed, intrinsic drug resistance is high in NTM, and multidrug treatment regimens must be given for at least 12 months with poor outcomes. In this context, the emergence of antimicrobial resistance (AMR) is of important concern during mycobacteria treatment, mainly due to its lengthy course and tough medical adherence, contributing to the projection to become one of the leading causes of death by 2050 [4, 5]. Therefore, there is an urgent unmet clinical need to develop complementary therapeutic modalities to fight AMR for both TB and NTM infections. The search for alternative therapeutic strategies relies on *in vitro* and *in vivo* models to better understand host–pathogen interactions and identify potential new therapeutic targets. Thanks to these models, the pathogenesis and immune evasion mechanisms of mycobacteria, especially Mtb, have been extensively studied and discussed in the literature (Table 1) [6, 7, 8, 9, 10, 11, 12], similar to their exploitation for new therapeutic approaches such as host‐directed therapies [13, 14, 15, 16, 17, 18, 19, 20].

**Table 1:** Mycobacteria pathogenesis summary.

	*Mycobacterium tuberculosis*	*Mycobacterium abscessus*
Type	Slow growing, obligate pathogen	Fast growing, opportunistic pathogen
Prevalence	In 2023, 10.8 million individuals fell ill (~1.25 million deaths)	3–6 cases per 100 000 persons
Risk factors	Undernutrition, HIV, type 2 diabetes, smoke	Cystic fibrosis, chronic obstructive pulmonary disease
Transmission	Person to person (Airborne droplets)	Environment (e.g., tap water)
Primary infection site	Distal airway (alveoli)	Proximal airway (bronchi/bronchioles)
Main cell target	Alveolar macrophages	Epithelial cells, and macrophages
Host immune response	Predominant Th1 response (IFN‐γ mediated)	Mixed, neutrophils and macrophages (pro‐inflammatory)
Immune evasion mechanisms	Inhibition of phago‐lysosome formation, disruption of phagosome maturation	Glycopeptidolipids‐mediated phagocytosis inhibition, biofilm and cording formation
Latency	Remain dormant in granulomas	No. Rarely observed in granulomas
Vaccine	Yes (BCG)	No
Treatment	Antibiotics (e.g., rifampicin, isoniazid, pyrazinamide, ethambutol)	Antibiotics (e.g., aminoglycosides, beta‐lactams, macrolides)
Upcoming challenges	Antimicrobial resistance (acquired)	Antimicrobial resistance (intrinsic)

**Table 2:** primer sequence for RT‐qPCR (related to Fig. 4).

Primer	Forward	Reverse	Role
SFTPB	TGGAGCAAGCATTGCAGTG	ACTCTTGGCATAGGTCATCGG	Surfactant, AEC2
AQP5	GCCACCTTGTCGGAATCTACT	GGCTCATACGTGCCTTTGATG	Aquaporine, AEC1
SCGB1A1	TTCAGCGTGTCATCGAAACCC	ACAGTGAGCTTTGGGCTATTTTT	Club Cells
DNAH6	CAGGAGGGCTTGTCGATTTG	CACGTCAGCCTCATGCAATAA	Ciliate Cells

Recent advances in stem biology offered the opportunity to model human tissues in a dish thanks to organoid technology. Even if human‐based animal‐free organoid models for mycobacteria infection modeling are at their infancy, in this review, we discuss the potential of the human lung organoid to implement the available *in vitro* and *in vivo* models for mycobacteria infection modeling and drug testing.

## The respiratory tract

The respiratory tract is a complex system composed of semi‐rigid conducting airways that originate from the trachea, bifurcate at the bronchi, and progressively narrow into the bronchioles. This anatomical arrangement culminates in the alveoli, where the exchange of respiratory gases occurs [21].

**Fig. 1:**
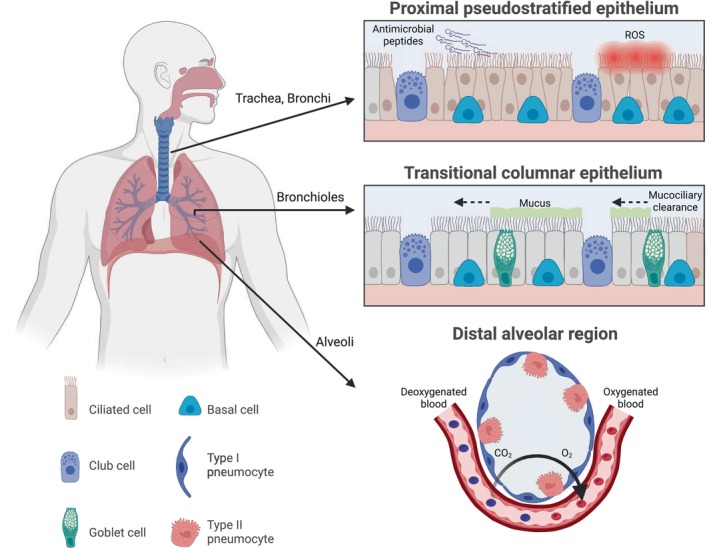
Cellular composition and defense mechanisms of the respiratory tract. The respiratory tract comprises three zones: proximal pseudostratified epithelium, transitional columnar epithelium, and distal alveolar region. All the zones are formed by specialized epithelial cells, which possess a broad range of defense mechanisms against pollutants and pathogens, such as antimicrobial peptides, reactive oxygen species (ROS), mucus, and mucociliary clearance. Created in https://BioRender.com.

The respiratory tract has evolved along the proximal–distal axis in a zoned manner, where a unique and diverse cellular composition characterizes each region [22]. In a reductionist way, the healthy human proximal tracheal and bronchial regions are formed by a pseudostratified epithelium composed of basal, ciliated, goblet, and serous cells. More distally, the bronchial area becomes a simplified columnar epithelium composed of ciliated, club, some basal, and goblet cells (Fig. 1) [23]. In the conducting airways, each individual cell has a specialized function. At steady state, the basal cells are considered the main stem cell population of the respiratory epithelium, contributing to tissue regeneration. The club cells secrete the surfactant liquid, which has immunomodulatory functions. Goblet cells generate mucus where pollutants and pathogens are trapped, while the ciliated cells are responsible for subsequently removing these through mucociliary clearance (Fig. 1) [24].

The conducting airways extend to an alveolar region, which is covered by alveolar type 1 (AEC1) and type 2 epithelial cells (AEC2) (also known as pneumocytes). AEC1 cells are flattened and squamous. These cells facilitate gas exchange in the lung and are associated with the endothelial capillary plexus, creating a delicate, gas‐permeable interface (Fig. 1). AEC2 are cuboidal and prevent the collapse of the alveolus during breathing by producing surfactant liquid. Additionally, upon injury, AEC2 contribute to the preservation of tissue integrity as they are considered progenitors of the AEC1 [25].

Apart from participating in gas exchange, the epithelial cells comprising the airways act as the initial barrier against inhaled pathogens and pollutants [26]. Among the principal defense mechanisms of the airway epithelium against pathogens are the barrier function, mucus production, mucociliary clearance, and activation of cell‐autonomous immune responses (Fig. 1). These immune responses encompass the production of antimicrobial peptides (AMPs), reactive nitrogen species, reactive oxygen species (ROS), growth factors, cytokines, and chemokines. Collectively, these mechanisms result in increased recruitment of white blood cells and communication with mesenchymal cells in the airway wall, enabling the airway epithelium to play a direct role in host defense and lung repair [27, 28, 29].

The mucus is an extracellular gel consisting of water, mucins, and various associated molecules, playing a crucial role in mucociliary clearance. Among the principal cells responsible for generating mucins are goblet, club, serous, and glandular mucous cells [27]. Mucins are large glycoproteins that carry multiple O‐glycans organized in central domains of tandem repeats abundant in serine and threonine [30]. Various factors, such as inflammatory cytokines, inhaled toxins, and microorganisms, trigger mucin gene expression. Following their production, mucins are stored in condensed granules until a stimulus (e.g., ATP, calcium) activates their release [27]. Among the main mucins secreted in the airway are MUC5B, MUC5AC, and MUC2 [31].

Mucociliary clearance is a complex process that takes place in the airway surface liquid, which consists of the periciliary liquid layer (PCL), cilia, and mucus. Contaminants and pathogens are trapped in the airway surface liquid mucus, preventing them from reaching the distal part of the respiratory tract. These trapped particles are then removed through coordinated regulation of ciliary movement. Furthermore, AMPs in the airway surface liquid, such as lactoferrin, cathelicidin, lysozyme, and defensins, contribute to the control of bacterial proliferation and facilitate the mucociliary clearance mechanism [23, 32, 33].

AMPs, typically consisting of 10–50 amino acids, are crucial components of the innate immune system in complex organisms. AMPs present extensive inhibitory effects against microorganisms such as bacteria, fungi, parasites, and viruses [34]. Airway epithelial cells (EC) generate diverse antimicrobial peptides such as β‐defensins, cathelicidins, lactoferrins, and lysozymes; each one of them mediates pathogen killing through different mechanisms [24]. Defensins are considered small cationic peptides (2–5 kDa) composed of a β‐sheet core and three intramolecular disulfide bonds that maintain their structure [35]. Human EC express, synthesize, and secrete four different types of β‐defensins. β‐defensin‐1 is constitutively secreted, while the others seem to be inducible. Indeed, they are produced in response to pro‐inflammatory cytokines or activation of Toll‐like receptors (TLRs) and nuclear factor‐κB pathways [27]. The main functions exerted by defensins are the lytic degradation of bacteria membranes [24] and the chemoattraction and activation of immune cells, such as dendritic cells [27]. Another important AMP generated by EC is cathelicidins. These peptides are stored in their preprocessed form within granules and are characterized by the presence of two hydrophobic α‐helix domains. Upon stimulation of the EC, the cathelicidin pre‐propeptides are cleaved at their N‐terminal domain resulting in their active form. So far, LL‐37 has been reported to be the only cathelicidin generated and secreted by human lung epithelial cells [36]. The antimicrobial effects of LL‐37 depend on its capacity to form pores in the cell wall of the mycobacteria and attract monocytes, neutrophils, and CD4^+^ T cells, as well as activate mastocytes [24, 37, 38]. Finally, the AMPs lysozyme and lactoferrin participate in maintaining lung homeostasis by controlling infections by gram‐positive and gram‐negative bacteria, respectively, through the degradation of their membranes. In addition, because lactoferrin is an iron chelator, it creates a hostile environment for bacterial growth [24].

ROS are well‐known signaling molecules generated by EC in response to injury. Moreover, recent evidence has recognized their function as direct antimicrobial agents through two mechanisms: disruption of lipid peroxidation at microbial membranes and DNA damage [39]. Both mechanisms rely on ROS production by EC through NADPH oxidases such as DUOX 1 and DUOX2 [27]. These ROS contribute directly to bacterial control in the airway surface liquid and serve as a precursor for the generation of antimicrobial molecules such as hypothiocyanite [40]. In addition to ROS, airway epithelial cells produce reactive nitrogen species via nitric oxide synthase enzymes (NOS‐1 and NOS‐2). The resultant nitric oxide collaborates in regulating the immune system and facilitating host defense against infections [41, 42].

## Human lung organoids for respiratory epithelium modeling

### Overview

**Fig. 2:**
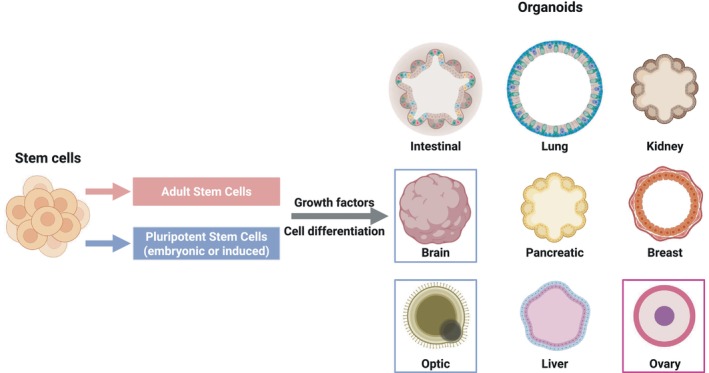
Human organoids are derived from stem cells. Human organoids are derived from adult (ASCs) or pluripotent stem cells (PSCs). The former recapitulate the main functions, architecture, and cellular components of mature adult human tissues, while the latter mimic developing tissue. Blue box: Organoids generated only from PSCs. Red box: Organoids generated only from ASCs. No box: Organoids generated from both. Created in https://BioRender.com.

Human diseases are challenging to study due to the complexity of biological processes. For this reason, scientists resort to model systems that range in complexity and scale from 2D cultures of single cells to animal models. While animal models are valuable for understanding physiology and pathophysiology *in vivo* and assisting in the preclinical development of treatments, they are expensive, difficult to interrogate, ethically challenging, and suffer inter‐species differences with human biology. As a result, three‐dimensional (3D) cell cultures, such as tissue‐specific epithelial‐based organoids, have emerged as attractive systems that reproduce *in vitro* fundamental characteristics of *in vivo* human tissue and organ complexity while being more experimentally tractable than *in vivo* models [43]. Organoids are 3D structures derived from pluripotent stem cells (embryonic (ESC) or induced (iPSCs)) or adult stem cells (ASCs) (Fig. 2) [44]. Pluripotent stem cells (PSCs) have the ability to differentiate into all the different cell types. In contrast, the ASCs are multipotent/unipotent, which restricts their ability to differentiate into a few cell types of the body [45]. Adult stem cells reside in fully differentiated tissues and are able to self‐renew, playing an important role in replacing dead cells and repairing damaged tissue [46]. ASCs are present in a broad range of tissues, such as skin, liver, pancreas, kidney, lung, bone marrow, fat tissue, among others. However, the number of ASCs and their renewing capacities diminish along with aging [45].

Organoids mimic the architecture and key functions of the epithelium from which they are derived, while maintaining their genetic stability for years [47, 48, 49, 50, 51]. It is important to note that when establishing organoid cultures, it is crucial to provide the stem cells with an optimal environment that supports their differentiation and self‐organization. This has been achieved using scaffolds and laminin‐rich extracellular matrices, as well as by providing the stem cells with a complex cocktail of growth factors and inhibitors [52, 53]. Interestingly, the same analytical techniques employed with single‐cell cultures can be applied to organoids, making them a useful model in basic research, disease modeling, and regenerative medicine [54, 55]. Moreover, organoid biobanking holds potential for drug screening and personalized medicine. Even though organoids partially recapitulate organ complexity (no vasculature, no innervation, no immune cells) [56], drug regulatory agencies such as the Food and Drug Administration have exploited the organoid technology in preclinical and clinical phases of new drug development to assess their toxicity [48, 57, 58]. Therefore, organoids bridge the gap between *in vitro* and *in vivo* models [59, 60], offering new opportunities to model infectious disease, especially by human‐restricted pathogens such as Mtb.

### Human lung organoids

Human lung organoids can be derived from either PSCs or ASCs. Regardless of their origin, these organoids mimic *in vitro* the 3D architecture (e.g., conducting airway, transitional respiratory airway, and alveoli) and essential functions (e.g., ciliary beating, and mucus and surfactant production) of lung epithelia [51, 61, 62]. To date, lung organoids have been applied to study chronic diseases [63, 64], such as idiopathic pulmonary fibrosis [65, 66], asthma [67], chronic obstructive pulmonary disease (COPD) [68, 69], primary dyskinesia [70], CF [51], and respiratory infections [71, 72, 73]. Actually, “human models” have become imperative to study the pathogenesis of respiratory infections, surpassing traditional mouse models [74]. The main reasons for this change in recent years rely on the differences in cellular composition between mouse and human respiratory tracts (e.g., absence of goblet cells in mice) [75, 76, 77]. These variations make the study of pathogens with human cellular tropism challenging [74]. Unlike murine models, lung organoids offer a promising alternative to study respiratory pathogen interactions with their host [78].

### Types of human lung organoids

The respiratory tract is an intricate zoned system, where each compartment is populated by various epithelial cell types [79]. Accordingly, lung organoids are classified into three groups: proximal lung organoids that replicate the conducting airway, intermediate lung organoids corresponding to the transitional respiratory airway, and distal lung organoids recapitulating the alveoli [79, 80]. Lung organoids are derived from stem and progenitor cells present in the lung tissue, including basal cells and secretory club cells in the proximal and middle airways, as well as AEC2 in the distal airways [81].

### Proximal lung organoids

The proximal lung organoids are comprised of the tracheospheres or bronchospheres. In 2009, Rock *et al*. [82] showed that murine and human basal lung epithelial cells can spontaneously organize into multicellular aggregates when cultured in a 3D air–liquid interface (ALI). These organoids could be passaged at least twice and consist of a P63‐basal cell layer and apical tubulin‐ciliated cells facing an inner lumen [51, 80].

However, these organoids still lacked certain cells that form the conductive airways. In 2015, Danahay *et al*. [83] using human bronchospheres as a model system, revealed that the Notch pathway has multiple actions in basal cell differentiation: blocking the Notch1 receptor increases basal cell markers, whereas blocking the Notch2 receptor increases ciliated markers at the expense of goblet markers. This advancement enabled another research group to develop a methodology to produce mature bronchospheres from human basal cells in 2016. The bronchospheres were comprised of ciliated, goblet, and basal epithelial cells [51, 84].

Although at the time, organoids showed to be a promising system in the development of more relevant models for the study of human diseases, they continue to face challenges in achieving long‐term expansion and fully replicating the complexity of the epithelial cell composition of the human lung [49].

### Intermediate lung organoids

**Fig. 3:**
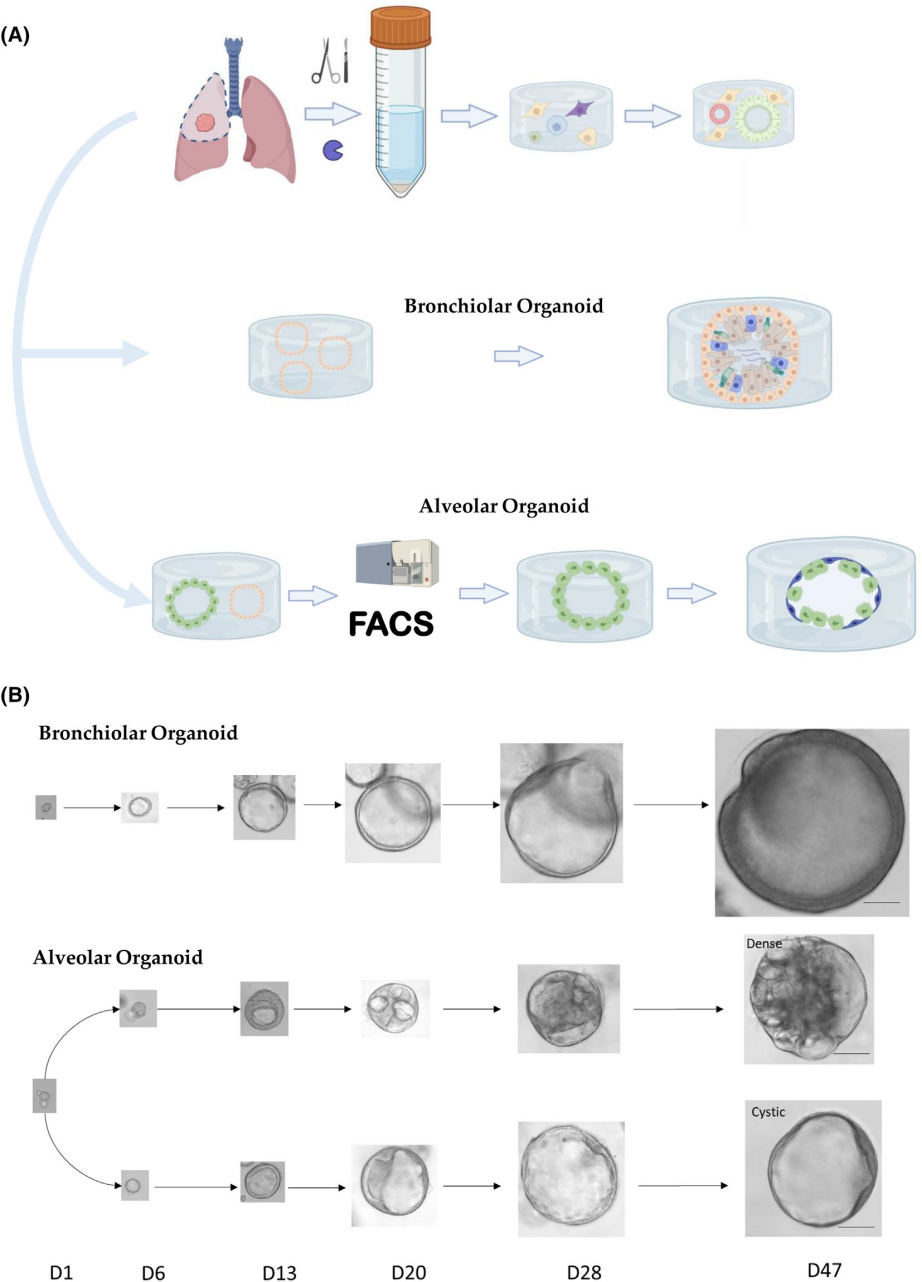
Bronchiolar/airway and alveolar organoid cultures. (A) Human lung organoids were derived from lung tissue resection obtained from the CHU of Toulouse (approved protocol CHU 19244 C/CNRS 205782). Human bronchiolar and alveolar organoids are generated from a single lung biopsy of human lung tissue, which is digested and seeded in a growth factor cocktail and an extracellular matrix as previously described [51, 64, 90]. In the case of alveolar organoids, after 1 month in culture, a single cell suspension was generated, alveolar type 2 epithelial cells (AEC2) were sorted using a BD FACSAria™ Fusion Flow Cytometer, and cultured as mentioned above. (B) Bronchiolar and alveolar organoids growth over time. Alveolar culture showed a mix between dense and cystic organoid structures. Scale bars = 100 μm.

**Fig. 4:**
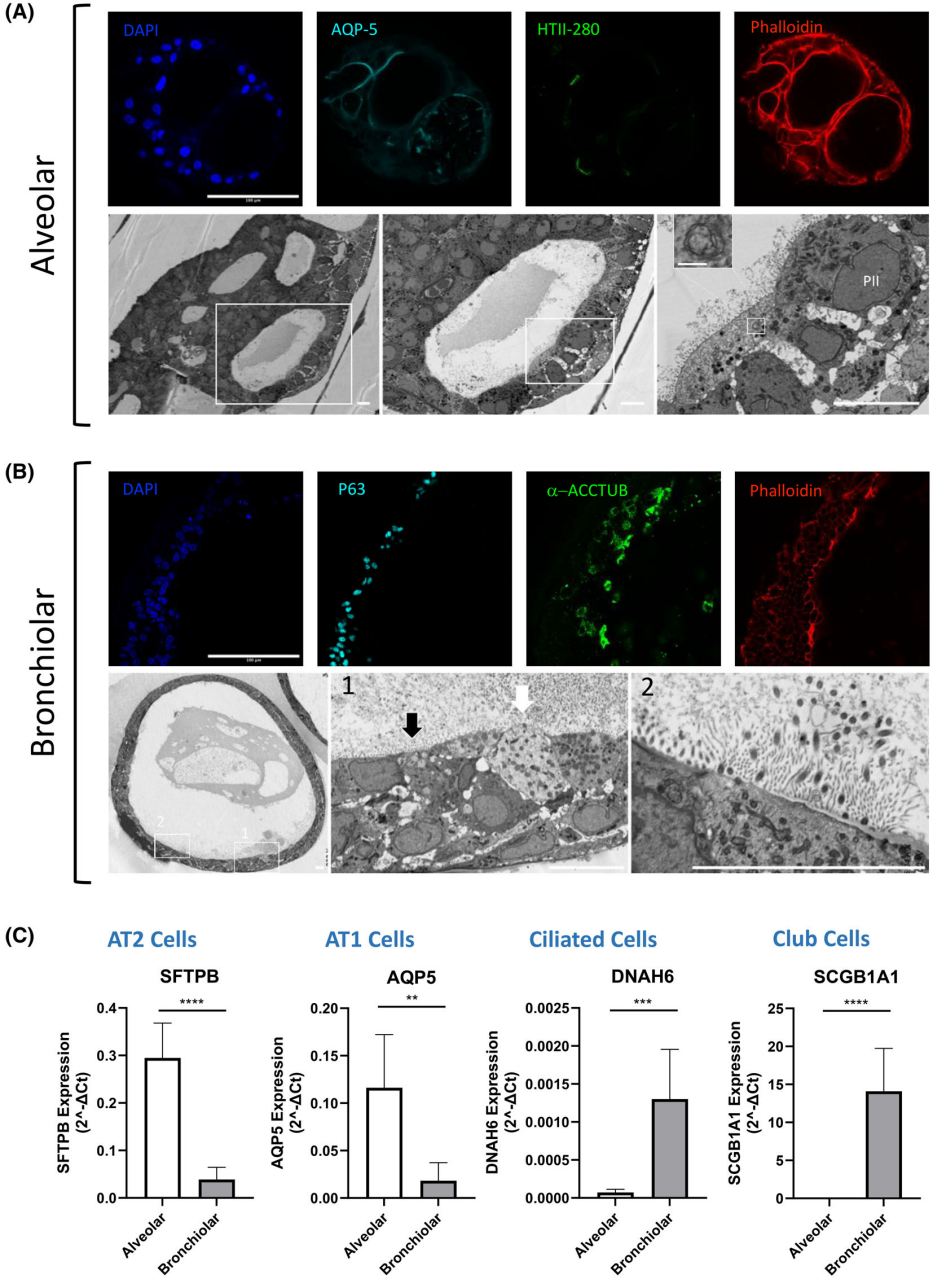
Characterization of bronchiolar/airway and alveolar organoids. Organoids were fixed (4% paraformaldehyde, Thermo Scientific Chemicals, Waltham, MA, USA), and immunostained as describe below. Nuclei and actin filaments were counterstained with DAPI (dark blue) and Alexa Fluor 546 Phalloidin (red) respectively. Organoids were imaged using a Zeiss LSM 710 microscope. For electron micrographs, samples were embedded in epoxy resin, thin sections were prepared as described in [111] and imaged at different magnifications on a SEM Zeiss Sigma 300 with a HDBSD detector. White boxes show the limits of the region of interest that was acquired at higher magnification in the next images. (A) The upper panel shows confocal images of an alveolar organoid stained for Aquaporin‐5 (AEC1 cells, light blue; Abcam ab92320, Cambridge, Cambridgeshire, UK) and HTII‐280 (AEC2 cells, green; Terrace Biotech TB‐27AHT2‐280, San Francisco, CA, USA). Scale bar = 100 μm. The bottom panel shows electron microscopy images of an alveolar organoid. The scale bar indicates 10 μm in the 3 panels. In the right panel inset image, scale bar = 1 μm. (B) The upper panel shows confocal images of a bronchiolar organoid stained for P63 (basal cells, light blue; Abcam ab124762) and acetylated alpha tubulin (ciliated cells, green; Santa Cruz Biotechnology sc‐23 950, Dallas, TX, USA). Scale Bars = 100 μm. The bottom panel shows electron microscopy images of a bronchiolar organoid. The arrows points to the cilia facing the lumen of the organoid. The scale bar indicates 10 μm in the 3 panels. (C) For RT‐qPCR analysis, organoids were lysed using RLT buffer (Qiagen, Hilden, North Rhine‐Westphalia (Nordrhein‐Westfalen), Germany), total RNA were extracted using the RNeasy Mini Kit (Qiagen) and reverse‐transcribed using the Verso cDNA Synthesis Kit (Thermo Fisher Scientific, Waltham, MA, USA). The expression of the target mRNA was assessed using the ABI 7.500 real‐time PCR system (Applied Biosystems, Foster City, CA, USA) and SYBRTM Select Master Mix (ThermoScientific). Differential cell markers expression of bronchiolar (DNAH6/ciliated cells, SCGB1A1/club cells) and alveolar (SFTPB/AEC2 cells, AQP5/AEC1 cells) organoids (Table 2). Graphs represent means ± SD from three pooled independent experiments, performed in triplicates. **P < 0.01, ***P < 0.001, ****P < 0.0001 by unpaired *t* test.

The intermediate organoids are constituted by the bronchiolar organoids, also known as airway organoids. In 2017, Emma Rawlins and co‐workers were the first to identify signaling pathways that allow long‐term self‐renewal of murine primary fetal lung tip progenitors as differentiation‐competent organoids [62]. The same year, Chen *et al*. [85] described the development of bronchoalveospheres from PSCs. They showed that, after 170 days of culture, the generated lung bud organoids display features of the second semester fetal lung, for which branching morphogenesis and proximo‐distal specification can be enhanced after 7 months of xenotransplantation in mice. Finally, they demonstrated that those organoids constitute a reasonable system for human disease modeling such as infectious disease (e.g., RSV infection) and fibrosis (by genetic manipulation of HPS1). It was not until 2019 that Hans Clevers and co‐workers developed a long‐term culture methodology for airway organoids derived from ASCs [51], which form after approximately 21 to 28 days in culture (Fig. 3). These organoids are fully differentiated and are composed of a polarized, pseudostratified airway epithelium containing club cells, ciliated cells, goblet cells, and basal cells. Importantly, both the secretory club cells and the cilia were functional, as evidenced by the beating of the cilia and the presence of mucus in the lumen of the organoids (Fig. 4). It is worth noting that human airway organoids can not only be developed from “healthy” adult tissue, but can also be generated from lung cancer or CF patient tissue, under the same culture conditions [51].

### Distal lung organoids

Belonging to the distal lung organoid group are the alveolar organoids. The alveolar epithelium shows two major cell types, the AEC1 and the AEC2 cells [79]. The cuboidal AEC2 function as tissue stem cells and are able to differentiate into AEC1 to restore tissue integrity after lung injury. They also secrete pulmonary surfactant, reducing surface tension, and alveolar collapse. The flattened AEC1 comprise the majority of the alveolar surface, directly ensuring gas exchange by close contact with capillary endothelial cells [86]. The first 3D alveolospheres were generated from murine AEC2 cocultured with fibroblast, mesenchymal cells or lung endothelial cells into an extracellular matrix. These alveolospheres consisted of AEC2 (SFTPC positive) and AEC1 (AQP5 and HOPX positive) cells [80, 86]. To date, fibroblast‐free long‐term 3D cultures of single adult human AEC2 or KRT5 positive basal cells have been developed by utilizing fibroblast‐expressed ligands and small molecule inhibitors [74, 87]. This was achieved through the magnetic bead‐based sorting of AEC2 cells using the apical surface marker HTII‐280 [68, 88, 89]. Additionally, a culture media containing activators of the WNT pathway, such as the molecule CHIR99021, was used to allow the expansion and maturation of AEC2 [90, 91]. The alveolar organoids form around 14 days (Fig. 3) and are comprised of exterior AEC2 and AEC1 facing the lumen of the structure (Fig. 4). These organoids can be long‐term expanded [92]. Alveolar organoids generated from primary sources have shown tremendous promise in mimicking disease pathology and addressing the scarcity of physiologically relevant *in vitro* models to test new therapeutic compounds [80, 93]. A recent example of this was the implementation of organoids to study SARS‐CoV‐2 infection [90, 92, 94, 95].

### Other lung organoid models

Organoids are a constantly evolving technology. Several other lung organoid models have been created and are worth mentioning. One example is the human nasal epithelial organoids (HNEO), which are generated from patient nasal brushing samples [96]. These organoids grow after 21 days of culture and are composed of basal cells, club cells, goblet cells, and ciliated cells [97]. HNEO shows differences in mucociliary cell composition and range of fluid secretion compared to airway organoids [98]; however, they constitute a valuable alternative for modeling diseases when access to lung biopsies or resection is not possible [70, 96, 97, 99, 100].

Finally, during the coronavirus disease (COVID‐19) pandemic, researchers generated a complete lung organoid model (also known as the bronchioalveolar model) derived from adult stem cells. This organoid model was developed from bronchial tree biopsies. Further surgical resection, the tissue was digested, and the cells were seeded in 3D and cultured for 10 days in a growth factor cocktail supplemented with Wnt3, R‐spondin, and Noggin. The complete adult lung organoid was scalable, propagable, personalized, and cost‐effective. The complete lung organoid showed both proximal (basal, ciliated, club, and goblet cells) and distal (AEC1 and AEC2) airway epithelia. This model revealed that proximal lung epithelial cells were fundamental for sustained SARS‐CoV‐2 viral infection, meanwhile distal alveolar cells were crucial in triggering an exacerbated immune response in fatal disease [94, 101, 102, 103, 104].

In summary, human lung organoids represent state‐of‐the‐art platforms recapitulating the main architecture and functions of the different epithelia of the human lung, and therefore, they are promising tools for the modeling of human lung diseases and drug testing [63]. With the emergence of new pathogens and the increase in antimicrobial resistance, new models are necessary to elucidate mechanisms of pathogenesis and to identify novel druggable pathways [72]. Until now, lung organoids have been used in studies of a number of viruses that cause respiratory diseases, including influenza and parainfluenza viruses, respiratory syncytial virus, and coronaviruses [73]. In contrast, further research on bacteria is needed; so far, there are very few bacterial studies using organoids: three with *Pseudomonas aeruginosa*, one with *Streptococcus pneumoniae*, and three with Mycobacteria [105, 106, 107, 108, 109, 110, 111, 112]. Yet, their potential for drug testing in the context of infectious diseases has to be thoroughly explored.

## Mycobacteria infections, for example, *Mycobacterium tuberculosis*

### Overview

*Mycobacterium tuberculosis* complex was identified as the causative agent of TB by Robert Koch in 1882 [113]. Mtb has evolved to become the perfect example of an obligate pathogen adapted to humans [114]. TB is heterogeneously distributed worldwide. However, the countries with higher numbers of TB cases reported are India, China, Indonesia, the Philippines, and Pakistan [115, 116]. Although TB is curable and preventable, in recent years, most of the new cases and TB‐related mortality have been associated with five primary risk factors: undernutrition, HIV infection, type 2 diabetes, alcohol use disorders, and smoking [116]. Of these, HIV infection is the most significant, accounting for 161 000 deaths among TB/HIV‐positive individuals in 2023 [116, 117]. The increased mortality in TB/HIV‐positive individuals may be a consequence of impaired communication between pro‐inflammatory alveolar macrophages and effector T cells, which promotes an anti‐inflammatory environment that allows Mtb to thrive [118]. The transmission of Mtb occurs predominantly through person‐to‐person contact via aerosolized microdroplets. Through coughing or sneezing, people with active pulmonary or laryngeal TB generate droplet nuclei (dried mucus droplets) that contain few viable Mtb [119]. Once Mtb is transmitted, between 5 and 15% of people will develop the active TB disease within months or years, while the remaining percentage of individuals may develop the active disease at some point during their lifetime [2]. The standard treatment for TB patients consists of an extended regimen (6–9 months) of four antimicrobials (rifampicin, isoniazid, pyrazinamide, and ethambutol) [120]. Successful treatment of drug‐susceptible TB patients has been reported in 88% of the cases [116, 121]. However, inadequate antibiotic regimens have allowed Mtb to develop resistance, leading to the emergence of multidrug‐resistant TB (resistance to rifampicin and isoniazid) and extensively drug‐resistant TB (resistance to rifampicin, isoniazid, bedaquiline, and/or linezolid). The emergence of drug‐resistant TB decreases treatment success by 39–68% [116, 120, 121, 122, 123, 124]. Consequently, antimicrobial resistance remains one of the major challenges to be further addressed in the fight against TB. Therefore, it is essential to identify new druggable molecular targets against Mtb. To achieve this, it is necessary to understand how Mtb bypasses the defense mechanisms of epithelial cells, which serve as the first line of defense in the respiratory tract, to establish chronic infections.

### Mtb interactions with respiratory epithelia

Throughout the infection process, Mtb encounters various pulmonary niches in the human lung. These niches can be extracellular upon entering the respiratory tract, where Mtb interacts with the airway epithelium and the alveolar region. Once the host's innate immune response is initiated, the Mtb niche becomes intracellular after being phagocytosed by the alveolar macrophages (AM) and establishes the primary infection [123]. At this point, infected dendritic cells (DC) and macrophages migrate to the lymph glands, where they activate T lymphocytes by presenting Mtb antigens to them. This process facilitates the recruitment of immune cells to the site of infection and promotes the formation of granulomas (organized structures formed by innate immune cells surrounded by T and B lymphocytes), where Mtb remains intracellular and latent for years [2, 125]. However, when the host's immune system declines drastically due to factors such as malnutrition, HIV, type 2 diabetes, aging/immune‐senescence, TNF blockade treatments, among others [126], granulomas grow in size and fail to contain Mtb. In this stage, the latent infection is reactivated (to active TB disease), resulting in the generation of necrotic granulomas, extracellular growth of Mtb, destruction of lung tissue, and subsequent dissemination of Mtb (lung cavitation stage of TB) [123]. To date, research has primarily focused on AM as the main targets of Mtb infection [127]. Recent reviews offer a comprehensive overview of Mtb pathogenesis from an immune perspective [6, 128, 129, 130]. However, our knowledge of the early events during Mtb infection in humans and bacilli interactions with EC is limited [131].

The EC are crucial at sensing the bacilli and alerting/interacting with immune cells, thereby contributing to the immune defense against the bacteria [125, 132]. EC express diverse pattern recognition receptors to detect and recognize microorganisms [132]. Mtb is sensed by EC through TLR2, TLR4, TLR9, NOD2, Dectin‐1, and complement receptor 3 (CR3), which then activate diverse signaling pathways, leading to the production and secretion of diverse cytokines (e.g., TNF‐α, IL‐6, IFN‐γ, IL‐27, GM‐CSF, MCP‐1, IL‐10, IL‐8, CXCL10) [15, 133, 134, 135]. The timely release of these immune mediators initiates the activation and recruitment of diverse leukocytes to the lung [136]. For instance, stimulation of EC by *Mycobacterium bovis* BCG induced IL‐8 and IL‐6 secretion and neutrophil recruitment toward the infected epithelia. This process leads to an increased presence of CD4^+^ T cell‐associated type 1 helper (Th1) and Th17 cells, which are essential for combating pulmonary TB [137]. Interestingly, in primary EC, type‐1 and type‐2 cytokines have been demonstrated to control the expression of several TLRs [138]. Additionally, in response to BCG infection, primary EC cultures produced the anti‐inflammatory cytokines IL‐10 and IL‐22 [132]. This suggests that EC play a significant role at maintaining the balance between inflammatory and anti‐inflammatory responses during mycobacterial infections.

In the context of Mtb infection, it has been reported that human EC secrete AMPs such as β‐defensin‐2, cathelicidin LL‐37, and hepcidin [136]. The first two control Mtb growth and have chemotactic activity. Specifically, β‐defensin‐2 chemoattracts memory lymphocytes and immature DC, while LL‐37 chemoattracts neutrophils [132, 139, 140]. On the other hand, hepcidin modulates the pool of extracellular iron, a crucial micronutrient for Mtb growth and survival [141, 142, 143]. In consequence, the airway epithelium, being the first site of exposure to Mtb, has an essential role to initiate and guide the subsequent immune response, playing an active role in disease progression and outcome [136].

In addition to pathogen detection and defense barrier, the airway epithelium can also be directly infected by mycobacteria, as has been shown *in vitro* in bronchial primary epithelial cells, and EC [132, 144, 145]. Even though EC are considered nonprofessional phagocytic cells and appear to be relatively insensitive to Mtb infection *in vitro* [131], some mechanisms of mycobacterial uptake by EC have been described. For example, it has been shown that some epithelial transmembrane receptors, such as integrins and Dectin‐1, interact with Mtb cell surface effector molecules, including the adhesins malate synthase, ESAT‐6, and heparin‐binding haemagglutinin (HBHA), which favor Mtb uptake [146, 147]. Once intracellular in EC, Mtb is localized in non‐acidic late endosomes, which in most of the cases, allow the bacteria to thrive [148]. Nevertheless, EC do not constitute a privileged replicative niche for Mtb [145].

In some cases, the early response generated by the EC is not sufficient to eliminate the pathogens that invade them [132]. To address this, EC have developed a range of mechanisms that enable them to interact with and respond to infected cells, as well as to alert immune cells following infection. It was shown that EC, after activation by Mtb‐infected AM and monocytes, generate antimycobacterial peptides, defensins, and S100‐family members that were not induced during the direct infection of the EC [131]. Recent studies have revealed that primary bronchial epithelial cells infected with Mtb in ALI cultures secrete pro‐inflammatory cytokines, chemokines, and tissue growth factors that attract neutrophils to the apical part of the infected cells [145].

Interestingly, *in vitro* studies have shown that EC express antigen presentation molecules of class II (MHC‐II) and the co‐stimulatory protein CD86 on their surface [149]. This MHC‐II appears to be functional and can stimulate T cells [150]. Apart from the innate immune competence of EC, the mucociliary function constitutes the first line of defense of the human lung [125]. However, it remains unclear how Mtb bypasses the EC mucociliary function to reach the alveoli.

In the alveolar microenvironment, Mtb interacts with the alveolar lining fluid composed of complement proteins, hydrolases, and surfactant proteins released by AEC2 cells. These components have the ability to alter the Mtb cell envelope, thereby facilitating the processing and further inactivation of diverse virulence factors, such as Mannose‐capped lipoarabinomannan and Trehalose dimycolate [125]. These changes further enhance the control of Mtb by professional phagocytes through the promotion of the fusion between the phagosome and the lysosome [151], and by neutrophils through the increase in the production of ROS and inflammatory cytokines [152]. Interestingly, it has been reported that the alveolar lining fluid from aged individuals, which contains high levels of oxidation and inflammatory mediators, favors Mtb adaptation and replication in alveolar epithelial cells [153].

Notably, AEC2 also play a direct role in controlling Mtb by sensing the bacilli lipoarabinomannan via TLR2, TLR6, and Dectin‐1, leading to the secretion of cytokines (IL‐8, IL‐1α, IL‐1β, IL‐6), AMPs (β‐defensin‐2, LL‐37), nitric oxide, and surfactant proteins [140, 144, 154, 155]. The result of these preliminary interactions in the alveoli will determine the Mtb clearance or the establishment of the infection [156, 157]. Few studies evaluate the early events of Mtb infection and interactions with epithelial cells *in vivo* using mouse models. Cohen *et al*. [158] show that secretion of IL‐1β by Mtb‐infected macrophages triggers signaling in non‐hematopoietic cells necessary for Mtb dissemination from the alveolar lumen to the lung interstitium, a critical step in Mtb infection establishment. This study highlights the importance of the cross talk between hematopoietic and non‐hematopoietic cells in the lung during the early steps of Mtb infection, hardly accessible in human settings.

### Modeling Mtb infection with lung organoids

**Fig. 5:**
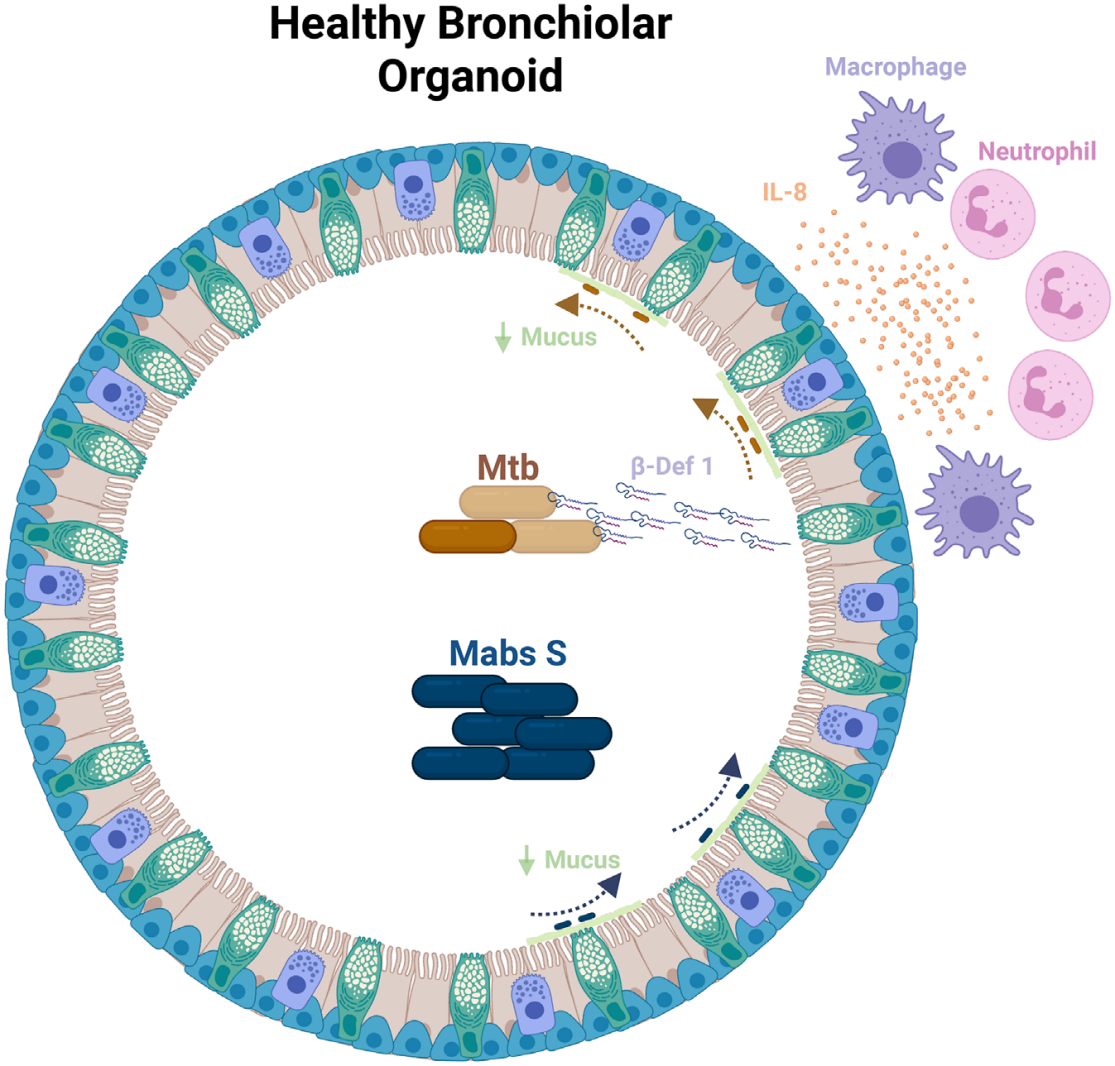
Bronchiolar/airway organoids response against mycobacteria infection. Healthy bronchiolar organoids controlled the growth of *Mycobacterium tuberculosis* (Mtb) through overexpression of cytokines (IL‐8) and antimicrobial peptides (β‐defensin 1), as well as by chemoattraction of immune cells such as macrophages. In contrast, the smooth morphotype of *Mycobacterium abscessus* (Mabs) thrived on organoid microenvironment and induced a weak organoid response. Both mycobacteria occupied mainly the lumen of the organoids as extracellular bacteria and interestingly, both downregulated the mucins expression by organoids during the infection. Created in https://BioRender.com.

Until now, few studies have adapted the lung organoid technology for mycobacteria infection modeling and drug testing [111, 112]. The investigation of early mycobacteria interaction with the lung epithelia is limited using *in vivo* models; the lung is not easily accessible, and the patients are often diagnosed at later stages of the infection. In addition, animal models, including mice, vary in epithelial cell composition between similar regions of the lung when compared to humans [132, 159]. The recent advances in organoid technologies offer a new opportunity to address this question. For the first time, in 2021, we implemented ASC‐derived airway organoid technology to study the interaction of Mtb with airway epithelial cells in human settings [110]. In this study, we showed that mycobacteria mainly reside as extracellular bacteria in the lumen of the airway organoids, with low efficacy to infect epithelial cells (Fig. 5). Mtb did not grow in the organoid lumen, consistently functioning as an intracellular pathogen. Interestingly, the airway organoids infected with Mtb exhibited overexpression of IL‐8 and β‐defensin‐1 and downregulated mucin expression, suggesting that Mtb could influence airway mucociliary function (Fig. 5). Finally, macrophages can interact with mycobacteria‐infected organoids, demonstrating that such organoid systems can be implemented with immune cells and offer a way to track innate immune cells and epithelium cross talk upon mycobacteria infection (Fig. 5) [110]. Therefore, mycobacteria‐infected organoids, as a preclinical model, offer a human 3D system to evaluate new antimicrobial therapeutic strategies against mycobacteria [160]. Recently, Kim and collaborators reported that Mtb can infect epithelial cells and macrophages in human pluripotent stem cell‐derived lung organoids. The infected organoids were effectively treated with classic anti‐TB drugs such as rifampicin and bedaquiline, and with host‐directed therapies [112]. In conclusion, the airway organoid technology is a valuable model to study the first interactions of mycobacteria with the airway epithelium. Future studies would have to further evaluate the potential impact of Mtb on the mucociliary function in the human airway, providing new insights on how Mtb reaches the alveolar space to establish its replicative niche. Moreover, airway organoids provide a relevant tool to evaluate new therapeutic strategies targeting Mtb in its extracellular and intracellular lifestyles. Finally, such approaches must now be extended to Mtb infection of alveolar organoid models to better reproduce the Mtb replicative niche in human settings [90, 94]. In this context, the introduction of resident immune cells such as alveolar macrophages is essential [161].

## Nontuberculous mycobacteria infections, for example, *Mycobacterium abscessus*

### Overview

Pulmonary infections caused by NTM are spreading throughout the world. More than 170 species of NTM have been documented, but only three of them, *Mycobacterium avium* complex, *Mycobacterium kansasii*, and *Mycobacterium abscessus* complex, are responsible for pulmonary infections in humans [162]. Contrary to TB, the prevalence of nontuberculous mycobacterial pulmonary disease is uncertain due to the lack of standardized diagnostic criteria. However, each year, the reported cases of nontuberculous mycobacterial pulmonary disease are rising in North America, Europe, and Asia [163, 164]. NTM infections are acquired through associations between the host, its environment, and the pathogen. Nevertheless, in the specific case of NTM, the most important factor for developing the infection is the presence of preexisting lung affections, such as previous pulmonary tuberculosis, bronchiectasis, COPD, primary ciliary dyskinesia, alpha‐1 antitrypsin deficiency, lung cancer, and CF [165, 166]. In patients with CF, nontuberculous mycobacterial pulmonary disease is observed in 6–13% of cases, with Mabs infection being particularly prevalent (ranging from 16 to 68%). *Mabs* is an opportunistic pathogen discovered in 1953 by Moore and Frerichs, who recovered the bacilli from a knee abscess of a 63‐year‐old female [167]. It is noteworthy that Mabs demonstrates a high degree of adaptation to the CF lung conditions [168]. NTM are mainly found in the environment [169]. However, in some hospitals, it has been confirmed that person‐to‐person transmission of Mabs (more specifically macrolide‐resistant strains) may be possible among CF patients [170, 171, 172].

Mabs is one of the most challenging NTMs to treat, as it is resistant to the majority of antibacterial molecules, including the regular treatments against tuberculosis [173]. As a consequence, treatment success in pulmonary infections caused by Mabs is very low, reaching a maximum of 50% efficiency [174]. The current treatment protocol for Mabs necessitates an 18‐month course of multidrug therapy [170, 175]. The British Thoracic Society recommends that the first 4 weeks of treatment comprise intravenous aminoglycosides (amikacin), tigecycline, beta‐lactams (cefoxitin or imipenem), and oral macrolide clarithromycin [175, 176]. After the first weeks of treatment, the continuation phase (14 months) begins, in which nebulized amikacin, oral macrolides, and additional antibiotics are used, such as linezolid, clofazimine, minocycline, cotrimoxazole, and moxifloxacin [177]. As Mabs treatment is long and tends to fail, new or repurposed treatments are needed [178].

### Cystic fibrosis, a risk factor for Mabs infection

CF is a disease affecting more than 160 000 people worldwide (estimated across 94 countries) [179, 180]. CF is a monogenic disease caused by mutations in the cystic fibrosis transmembrane conductance regulator gene (CFTR). Until now, more than 1700 mutations in this gene have been described [181]. The CFTR gene is located on chromosome 7, spans 250 kb, and is composed of 27 exons [182]. CFTR encodes an anion channel, activated by cAMP‐dependent phosphorylation, whose primary function is the transport of chloride and bicarbonate. In addition, the CFTR modulates the function of the epithelial sodium channel (ENaC) and regulates the water exchange across the apical plasma membrane of EC [179, 183].

Mutations causing CF are organized into seven classes depending on their functional defect. Class I mutations are due to premature truncation or nonsense alleles, thus often leading to degradation of mRNA and affecting protein production. Class II mutations trigger aberrant folding of the CFTR protein, causing its retention in the endoplasmic reticulum and posterior proteasomal degradation. This group includes the most common ∆F508 CFTR mutation, which is present in 70% of defective CFTR alleles and in 80% of patients with CF in Europe [184, 185]. Class III mutations lead to a full‐length CFTR channel that is incorporated into the cell membrane, but with impaired gating. Class IV mutations cause a decrease in CFTR channel conductance of chloride and bicarbonate ions [186, 187]. Class V mutations are due to alternative splicing, promoter, or missense mutations leading to a reduction at the surface of EC of the CFTR protein. Class VI mutations destabilize CFTR at the cell surface, resulting in enhanced endocytosis of the protein, its degradation at the lysosome, and impaired recycling to the cell surface. Lastly, class VII mutations are the most severe as they completely abolish mRNA transcription. These mutations cannot be rescued by pharmacological treatments [179, 185, 188].

Defective CFTR affects multiple systems, including the respiratory, gastrointestinal, glandular, and reproductive ones [189]. However, the principal system affected is the respiratory system. Among the consequences of an abnormal CFTR in the airways are the dehydration of the mucus, impaired mucociliary clearance, mucus accumulation, inflammation, oxidative stress, release of cytokines and chemokines (IL‐8, GM‐CSF), recruitment of neutrophils and macrophages, and chronic infections by opportunistic pathogens, such as Mabs [190, 191, 192, 193, 194]. In the past, the care for CF patients consisted of symptomatic treatments, including antibiotics against infections, mucolytics to reduce sputum viscosity and stickiness and improve expectoration, and finally physiotherapy to help reduce airway obstruction. However, recent advances in CF treatments allow the inclusion of etiologic therapies such as CFTR correctors and potentiators [195].

In recent years, it has become clear that patients with CF are more prone to colonization and infection by Mabs than other NTM [196]. Prospective and multicenter investigations have shown that CF is a major risk factor for NTM infections, with Mabs being the most prevalent [197, 198]. Mabs infection has been linked to decreased lung function in CF patients, thus being more likely to require a lung transplantation. However, CF patients with pretransplant Mabs infection are at risk to develop posttransplant invasive Mab disease. Consequently, Mabs are considered a relevant cause of morbidity and mortality in CF patients [199]. Furthermore, in an age‐related prevalence study, Mabs was isolated from airway secretions in all ages, in contrast to *Mycobacterium avium* complex, which was only observed in patients over the age of 15 [200]. Although it is well accepted that CF predisposes to Mabs infection, it is still unclear why. Recently, some studies have shown that CF patients are prone to colonization by bacteria that form biofilm, such as Mabs smooth (S) strains expressing glycopeptidolipids (GPLs), due to their altered pulmonary physiology [1].

Over the last decade, zebrafish have emerged as a valuable animal model to study Mabs infection. This model allowed progress in understanding Mabs dissemination, its pathogenesis, and the role of CFTR during infection [201, 202, 203, 204, 205]. However, despite these advances, the zebrafish model presents some important limitations, such as the lack of lungs and the absence of a complex respiratory epithelium [206]. Therefore, further research is needed to better understand Mabs colonization of the CF human lungs and improve the treatments against Mabs.

### Mabs interactions with respiratory epithelia

Mabs is the most virulent rapid‐growing mycobacterium. Nevertheless, the mechanisms employed by Mabs to colonize the lung and establish the infection are poorly understood. Contrary to Mtb, Mabs displays two distinct morphotypes: a smooth (S) variant and a rough (R) variant. The first is motile, biofilm‐forming, and unable to generate cording structures, while the second is nonmotile, cord‐forming, and non‐ or hyper‐aggregative/stiffer biofilm‐forming. Besides these differences, the key distinction between the S and R Mabs variants lies in the quantity of GPLs on the surface of their membranes. Specifically, the R form shows a notable reduction in surface‐associated GPLs [207, 208, 209].

It is known that GPLs play an important role in Mabs biofilm formation and further lung colonization [196]. Indeed, it has been reported that in patients with lung diseases, the GPL‐expressing S variant is capable of generating biofilms [210]. GPLs not only play a role during the biofilm formation but are also important for the sensing of Mabs by the EC. GPLs are immunologically inert and can mask bioactive molecules from the bacterial cell wall. Nevertheless, the loss of GPLs unmasks underlying Mabs cell wall molecules such as PIM, which are recognized by TLR2 on EC, leading to their activation and release of IL‐8 and human β‐defensin‐2 [211, 212]. Due to the fact that in the early stages of the infection the S variant predominates, Mabs partially eludes the innate immune system [1]. Interestingly, despite having GPLs on its cell wall, Mabs S showed interaction with normal human bronchial epithelial cells (NHBE), resulting in the overexpression of genes such as DUOX2, an enzyme involved in ROS production, and IL‐32, a cytokine associated with epithelial cell apoptosis [213]. It is worth noting that Mabs S was able to lower the percentage of epithelial ciliated cells *in vitro* as well as the ciliary beat frequency [213]. However, the slight increased expression of some genes, overall, denotes a low responsiveness of lung epithelial cells to the bacterial challenge [213, 214]. In contrast to Mabs S, NHBE overexpressed IFN‐I proteins (OAS1, Mx1, ISG15) and upregulated pro‐inflammatory (IL‐36) and chemoattractant (CCL5, CCL22) genes after being exposed to Mabs R cell wall microparticles [215]. Lastly, Mabs S revealed low internalization and survival in A549 cells [216]. In conclusion, the interactions between epithelial cells and Mabs will determine the appropriated sensing and clearance of the bacteria, and its further interaction with immune cells such as neutrophils, macrophages, and DC at the lung.

### Modeling cystic fibrosis‐associated mycobacteria infection with lung organoids

In 2013, Dekkers and collaborators modeled for the first time CF using organoids. In this study, CF patient‐derived intestinal organoids were generated and characterized. These organoids recapitulated the main hallmarks of the CF disease *in vitro*. On the other hand, in the same study, developed and applied the functional forskolin‐induced swelling assay was developed and applied. During this assay, healthy organoids react to cAMP stimulation (forskolin–cAMP signaling‐induced CFTR channel activity) by importing fluid into the lumen, inducing organoid swelling, easily trackable microscopically. Due to CFTR loss of function, CF patient‐derived organoids do not swell [217]. This *in vitro* assay allows, in a personalized way, stratification of CF patients responding to, and therefore being treated with CFTR modulator therapies [60, 218].

More recently, other organoid models have been created for CF study, such as the airway organoids and the HNEO [51, 96]. The CF airway organoids can be generated from patient lung resection (after lung transplantation) [111] or broncho‐alveolar lavage fluids [51]. Interestingly, airway organoids derived from CF patients carrying the F508del mutation exhibit secondary phenotypes such as mucus buildup and abnormal forskolin‐induced swelling [219]. These characteristics make airway organoids an accurate model for studying CF lung disease [51, 111]. Importantly, as previously reported in intestinal organoids, CF airway organoids treated with CFTR modulators in part rescue defective forskolin‐induced swelling [51]. On the other hand, CF HNEO were generated relatively easily from nasal brushings. These organoids showed a narrow lumen area and defective swelling in comparison to non‐CF controls. Swelling in CF HNEO derived from F508del CF patients was rescued after treatment with CF modulators [99, 220, 221].

We applied the human airway organoid technology to model Mabs infection in a CF environment. We showed that CF‐derived airway organoids have a thicker epithelium, accumulate mucus, undergo oxidative stress, and increase lipid peroxidation and cell death, all of which are key features of CF [111, 222]. Both Mabs S and R replicated more efficiently in CF patient organoids compared to the healthy ones. Remarkably, while Mabs S forms biofilms, Mabs R forms cords and displays a higher virulence, further validating the relevance of CF organoids as a model for the study of Mabs pathogenesis. Finally, we showed that boosting antioxidant pathways in combination with the antibiotic cefoxitin mitigates oxidative stress in CF airway organoids and supports better control of Mabs infection. Therefore, CF patient‐derived airway organoids also constitute a promising preclinical tool to evaluate not only innovative host‐directed strategies [111, 222] but also new antimicrobial compounds targeting Mabs [223].

## Advantages and limitations of the human lung organoid models

Human lung organoids are an advantageous model for studying respiratory infections compared to classic cell cultures and animal models. They are sophisticated multicellular systems that mimic the architecture and essential functions of the different parts of the human lung. Also, they can be cryopreserved in biobanks and remain genetically stable for years [224, 225]. Human lung organoids are an easily tractable model in comparison with animals, as well as reliable and scalable. As a result, the organoids have contributed in the last years to the progress of personalized medicine. Despite their numerous benefits, organoids have certain limitations, including lack of vascularization and immune cells, absence of an air‐liquid interface due to their cystic form, and enclosed architecture. This limits access to the apical epithelial surface, where most pathogens are initially sensed by host cells [60, 63, 226]. Nevertheless, the organoid field is still evolving. Recent advances such as the improvement of coculture protocols between organoids and immune/stromal cells [227], the generation of organoid‐derived ALI cultures with accessible polarized pseudostratified epithelium [51, 228], the development of the organoid‐on‐a‐chip technology replicating the circulation in the human body [229, 230], and the implementation of microinjection techniques and generation of apical‐out organoids [55, 231] have been adopted to overcome organoid limitations.

## Conclusion

In this review, we summarize the current knowledge on the role of the EC during the early stages of mycobacterial infections. The EC plays a crucial role in determining the outcome of the infection, either by direct clearance of the bacteria through cell‐autonomous defense mechanisms or by cross talk with other immune cells, such as macrophages. Despite the relevance of the EC in controlling mycobacterial infections, there are just a few studies focused on them. In most of the cases, this lack of research is due to the absence of reliable and physiologically relevant models to study the lung epithelia.

To address this gap, in this review, we introduce the lung organoid models as a promising technology to improve our understanding of host‐mycobacteria interactions in the respiratory tract and to decipher new treatments. Although few studies evaluated the potential application of lung organoids in the study of mycobacterial infections, this technology looks promising and could help address unresolved questions in the mycobacteria field, including mycobacterial entry, epithelial immune evasion mechanisms, adaptation to lung luminal niches (e.g., mucus content, hypoxia, and oxidative stress), regulation of mucociliary function and epithelial barrier integrity for bacteria dissemination, and biofilm formation. As NTM infection constitutes a clinical challenge not only for patients with CF but also for patients with non‐CF bronchiectasis, the non‐CF patient‐derived organoids could be implemented by accessing nasal cells as previously described for primary ciliary dyskinesia [70] and COPD [100]. By combining innate immune cells such as alveolar macrophages [232], the replicative niche for mycobacteria, with human lung organoids, these models could be implemented to test innovative therapeutic strategies targeting intra‐ and/or extracellular bacteria as well as host responses, as a complementary approach to classical human cell lines or animal models [111, 223, 233] to fight antimicrobial resistance in mycobacteria.

## Conflict of interest

The authors declare no conflict of interest.

## Author contributions

SAL‐I and CC: conceptualization, visualization, and original draft writing; SAL‐I, RV, and RM bronchiolar and alveolar organoid characterization, LB electron microscopy analysis, all authors edited and approved the manuscript.

## Data Availability

The data that support the findings of this study are openly available in Mendeley data at http://doi.org/10.17632/5p5hz4rj5y.1, reference number DOI: 10.17632/5p5hz4rj5y.1.
